# Meta-analyses of fertility desires of people living with HIV

**DOI:** 10.1186/1471-2458-13-409

**Published:** 2013-04-30

**Authors:** Yifru Berhan, Asres Berhan

**Affiliations:** 1 Hawassa University College of medicine and health sciences, Hawassa, Ethiopia

**Keywords:** ART, Fertility desire, Meta-analysis, HIV positive, Sensitivity analysis

## Abstract

**Background:**

Literature review has shown that some years back the fertility desires of people living with HIV was low but in the recent years, it was reported as increasing. However, little is known about the strength of association of fertility desire of HIV positive people with antiretroviral therapy (ART) experience, age, sex, education level, and number of children.

**Methods:**

In these meta-analyses, twenty studies from different parts of the world were included. The odds ratios of fertility desires were determined using the random-effects model. Heterogeneity among the studies was assessed by computing values for Tau^2^, Chi-square (Q), I^2^ and P-value. Sensitivity analysis and funnel plot were done to assess the stability of pooled values to outliers and publication bias, respectively.

**Results:**

The pooled analysis demonstrated that fertility desires of study participants had no association with ART. Similarly, the overall odds ratio did not show statistically significant association of fertility desires with sex and educational attainment of study participants although forest plots of some studies fall on increased and some others on decreased sides of fertility desires. The two variables that demonstrated a strong association with fertility desires were age less than 30 years and being childless. The lowest heterogeneity was found in a meta-analysis comparing ART experienced and ART naïve HIV positive people. In all meta-analyses, the sensitivity analyses showed the stability of the pooled odds ratios; and the funnel plots did not show publication or disclosure bias.

**Conclusion:**

Although the fertility desires among childless and younger age group was very strong, we realized that quite a significant segment of HIV-infected people have desire for fertility. Therefore, including fertility issue as integral part of HIV patient care may help several of them in their reproductive decision making (letting them know the risks and methods of prevention while anticipating pregnancy).

## Background

Before the era of HIV, wishing to have a biological child and preserving the continuity of their family to the future generation is perhaps a common thought of all and this is also expected to be same in these days in the majority of individuals who are sero-negative for HIV. However, several studies have shown that the fertility desire of people living with HIV was found to be significantly reduced [1-4]. A national survey in Cameroon revealed that women’s fertility desire was about 55% and the main factor independently associated with this desire was having good physical health [5]. In other African countries, the fertility desires were 15% in Malawi [6], 18% in Uganda [7], 41% in Ethiopia [4] and 63% in Nigeria [8]. One of US studies also showed that the majority of HIV positive women did not have desires to be pregnant [9].

On the other hand, the fertility desire of HIV-infected individuals were reported as increasing because of increasing hope of living longer with ART, resumption of a healthy life, and the overall physical and mental well-being. A multivariate analysis from Uganda showed that the fertility desire of HIV-infected individuals had significantly increased with ART [10]. In Canada, the proportion of fertility desire among HIV positive women increased from about 26% in 2007 [11] to 69% in 2009 [12]. Since majorities of HIV positive women are in the reproductive age, a decade back the fertility desire was also predicted that many will continue to have desire for children [13]. This is because; parenthood, particularly among married couples, is an important component of their social status. As noted by two qualitative studies (South Africa and Zimbabwe), the availability of ART and prevention of perinatal HIV transmission has also positively affected childbearing desires [14,15].

Several non-comparative studies have also shown the independent association of other factors with fertility desire in HIV-infected population. A partner’s desire for a child was one of the strongest predictors for women’s fertility desire [16]. In another study, being younger age, having fewer living children and higher quality of life were individually associated with fertility desire [17].

In general, a systematic review including twenty nine studies has also outlined the presence of many more factors that are related to fertility desires [18]. However, by design, it was not a quantitative or pooled analysis to more powerfully estimate the true effect size. Furthermore, from previously published primary studies, it was noted that the association of fertility desire with ART, age, sex, education level, and number of children were inconsistent. Thus, the objective of this pooled analysis was to demonstrate the strength of association of these variables with fertility desire.

## Methods

### Search strategy

Computer based search for articles related to the fertility desire of HIV positive people was conducted in Medline, HINARI, google scholar and Cochrane library. Via HINARI, we have also searched the websites of major publishers like: Elsevier Science-Science Direct, Nature Publishing Group, Oxford University Press, PsycARTICLES, Science and Wiley-Blackwell. The search was supplemented by searching the reference lists of each retrieved article. Article search was performed by both authors (YB and AB) with an alternate combination (and/or) of these search terms: “fertility desire”, “fertility intention”, “desire to have children”, “parenthood”, “motherhood”, “maternity paternity”, “people living with HIV”, “HIV” and “ART” or “HAART”.

### Study selection

These meta-analyses involved studies with the following inclusion criteria: 1) studies which reported the fertility desire of HIV positive individuals in relation to one or more of the selected variables (ART experience, number of children they have, age, sex and level of education); 2) studies which were published in English; and 3) studies done from year 2000 up to June 2012. Studies were selected if they met any of these variables.

### Data extraction

From the selected studies, the following information was abstracted: author, year of publication, country where the research conducted, sample size, number of HIV positive people having fertility desires (ART experience, number of children, age, sex, and level of education). Some variables were dichotomized as no child vs one and more, age less than 30 vs 30 years and above, primary or no education vs secondary and above. For these analyses, data on both fertility intention and desire were similarly entertained.

### Statistical analysis

Five meta-analyses were done taking ART experience, age, sex, education level, and number of children as independent variables. The odds ratios of fertility desires were determined using the DerSimonian-Laird method (Random effects model). Heterogeneity among the studies was assessed by computing values for Tau^2^, Chi-square (Q), I^2^ and P-value. When the value of I^2^ was greater than or equal to 50%, the variation across the studies was considered as statistically significant. Sensitivity analyses were done to assess the stability of pooled values to outliers. Publication/disclosure bias was evaluated by funnel plot. All the analyses were done using Meta Analyst (Beta 3.13) software [19]. P-value < 0.05 was considered as statistically significant.

## Results

**Figure 1:**
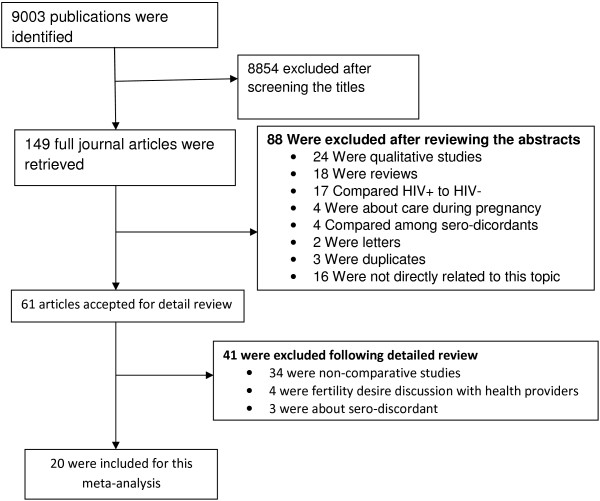
Flow diagram showing the process of study selection.

As shown in Figure 1, for the selected search terms, it was possible to access 9003 articles. Of which, 8854 were excluded after screening the titles for their relevance to these meta-analyses. Some others were excluded because of non-comparative nature of the studies; being qualitative by design or reviews; made a comparison of fertility desires between HIV positive and negative individuals or among sero-discordant. Finally, 20 studies (one each from Brazil, Canada and France, two from US and the rest from Africa) were eligible [7,10,20-37]. The general information on the included studies is presented in Table 1.

**Table 1:** General characteristics of studies included in these meta-analyses

**Author**	**Year**	**Country**	**Target**	**Study design**
Andia I et al.	2009	Uganda	Women	Cross-sectional
Cooper D et al.	2009	South Africa	Men & women	Cross-sectional
Erhabor O et al.	2012	Nigeria	Men & women	Cross-sectional
Finocchario-Kessler S et al.	2010	USA	Women	Cross-sectional
Heard I et al.	2007	France	Men & women	Cross-sectional
Iliyasu Z et al.	2009	Nigeria	Men & women	Cross-sectional
Kaida A et al. study 2	2010	South Africa	Women	Cross-sectional
Maier M et al.	2009	Uganda	Women	Cross-sectional
Nobrega A A et al.	2007	Brazil	Women	Cross-sectional
Myer L et al.	2007	South Africa	Men & women	Cross-sectional
Nakayiwa S et al.	2006	Uganda	Men & women	Cross-sectional
Paiva V et al.	2007	Brazil	Men & women	Cross-sectional
Stanwood N L et al.	2007	USA	Women	Cross-sectional
Alemayehu B et al.	2012	Ethiopia	Men & women	Cross-sectional
Getachew M et al.	2010	Ethiopia	Men & women	Cross-sectional
Kaida A et al. study 1	2011	South Africa	Women	Cross-sectional
Kipp W et al.	2011	Uganda	Men& women	Cross-sectional
Ogilvie G S et al.	2007	Canada	Women	Cross-sectional
Tamene W et al.	2007	Ethiopia	Men & women	Cross-sectional
Tesfaye L et al.	2012	Ethiopia	Men & women	Cross-sectional

**Figure 2:**
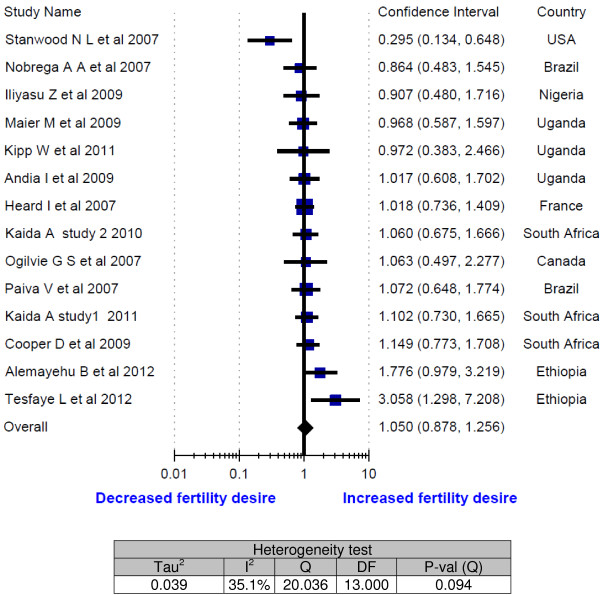
Meta-analysis of fertility desires of people living with HIV (Odds ratios of ART experienced vs ART naïve).

As presented in Figure 2, out of fourteen studies included in this meta-analysis [10,20-32], twelve showed no statistically significant association of fertility desires with ART. The two studies, one showed a statistically significant increment [31] and another one reduction [32] in fertility desire with ART. As a result, the overall odds ratio demonstrated that the fertility desires of study participants were not influenced by ART (OR = 1.1; 95% CI: 0.88 - 1.26). The testing for heterogeneity did not show much variability among the included studies (I^2^ = 35.1%).

**Figure 3:**
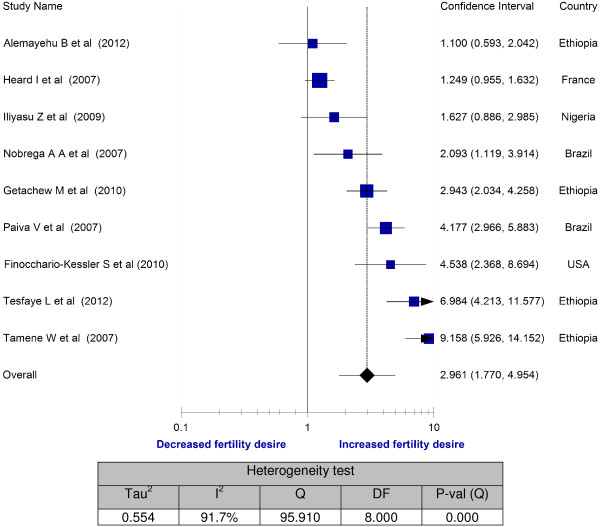
Meta-analysis of fertility desires of people living with HIV (Odds ratios of no child vs one or more child).

However, having no child was a strong predictor of fertility desire [20,28,31,33,34,37] (Figure 3). Although statistically significant associations were not seen in all studies, the majority of the study participants had a tendency for fertility desire as evidenced by the forest plot of all studies falling on the side of increased fertility desire. The pooled odds ratio also showed a significant association of fertility desire with HIV positive individuals who had no child (OR = 2.9; 95% CI: 1.77 - 4.95). The sensitivity analysis attested to the stability of the overall odds ratio. However, it should be noted that there was significant variability among the included studies (I^2^ = 91.7%).

**Figure 4:**
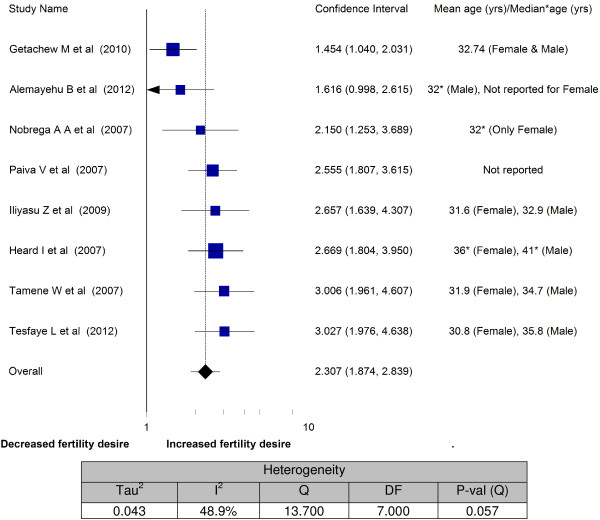
Meta-analysis of fertility desires of people living with HIV (Odds ratios of < 30 years vs 30 years and above).

In Figure 4, another predictor for fertility desire was the age of people living with HIV. The high fertility desires among age category below 30 years was almost a consistent finding in all included studies [20,21,26-28,31,33,34]. The fertility desires of this age category were about 1.5 to 3-fold higher than their older counterparts. As a result, the overall odds ratio demonstrated more than 2-fold increment in fertility desire in less than 30 years of age (OR = 2.3; 95% CI: 1.87 - 2.84). The heterogeneity testing showed moderate variability (I^2^ = 48.9%).

**Figure 5:**
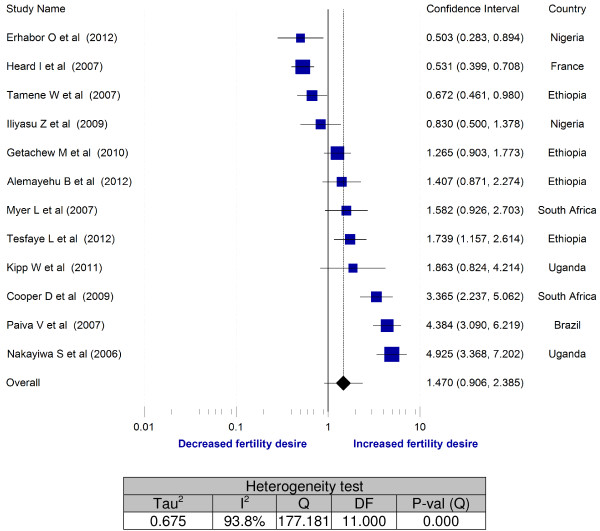
Meta-analysis of fertility desires of people living with HIV (Odds ratios of male vs female).

In Figure 5, pooled analysis was done for association of sex with fertility desire by including twelve studies. In four studies, men had more fertility desires than women [7,20,22,31]. Other three studies showed that being woman was found to have a statistically significant association with fertility desire [26,34,36]. In other five studies, fertility desires were not associated with sex of respondents [21,25,27,33,35]. The overall odds ratio also did not show statistically significant association of fertility desire with being women or men. There was significant variability among included studies (I^2^ = 93.8%).

**Figure 6:**
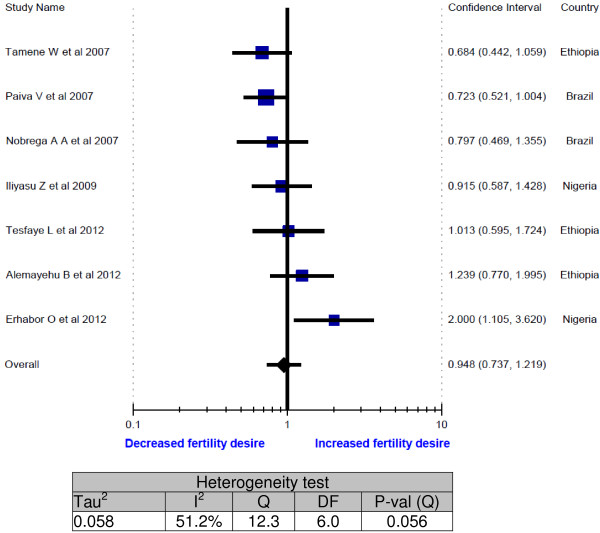
Meta-analysis of fertility desires of people living with HIV (Odds ratios of primary or no education vs secondary and above).

Except one study [36], the meta-analysis of fertility desire in relation to education level did not demonstrate a strong association of fertility desire with better education (Figure 6). In four studies, the forest plot fall on the decreased fertility desire side [20,27,28,34] but the pooled odds ratio was insignificant. In all these five meta-analyses, the funnel plot did not demonstrate publication or disclosure bias.

## Discussion

These meta-analyses demonstrated that the fertility desire was highest among young and childless people living with HIV but was not associated with ART or educational level. ART is known to improve the overall well-being of HIV-infected persons [38] and many speculated that better physical health and cognitive improvements gained due to ART can improve their positive attitude towards childbearing. Similarly, a better educated people are expected to have better awareness about preventive methods of mother-to-child transmission of HIV, and as a result, their fertility desire is anticipated to be higher than the less educated individuals.

However, as these meta-analyses showed, neither their better education attainment nor being on ART was found to have an influence on their fertility desire. Rather, fertility desire was strongly associated with being young or childless, which both were likely to be interlinked one with the other and this kind of reflection is probably the reflection of the majority of human being in the fertile age regardless of HIV infection. In another study not included in this meta-analysis, being young or having few or no child was also an independent predictor for high fertility desire [16]. To the contrary, in another study, being young or single was reported to be inversely associated with fertility desire [39]. These reports showed how inconsistent the fertility desire behavior of the study participants was, which is calling for further in-depth investigation.

In general, it is a known fact that identifying the factors behind the fertility desires has paramount importance for both policy makers and health care providers. Specifically, although these meta-analyses did not demonstrate the associations of fertility desire with ART, as majority of HIV positive people are in the fertile age and living longer with ART [40,41], the authors speculate that their fertility desire is going to increase as the duration of ART advances. Unlike the previous times when high HIV prevalence in many Sub-Saharan African countries was associated with about 20%-40% reduction in fertility [42], we are observing that these days the number of pregnancies among HIV positive women is increasing, which can be taken as a proxy indicator of increasing fertility desire among people living with HIV. However, as previous studies pointed out, the fertility issue does not seem given much emphasis in the package of HIV patients follow up and care [15,22].

This is despite the fact that fertility desires of people living with HIV are serious concerns for the patients themselves, for authors in the field and health care providers [14,15,40,43]. This is because; from the perspective of preventing HIV transmission and socioeconomic consequences, the implication of fertility desire is multifaceted. Because of its unprotected nature of the sexual practice, there is a high chance of HIV horizontal transmission to HIV-negative and/or positive individuals (a different HIV strain, probably even resistant ones). Secondly, if the fertility desire becomes successful and pregnancy occurs, there is high concern and increased risk of HIV vertical transmission to the coming baby during pregnancy and breastfeeding [4,40]. Thirdly, HIV infection is known to increase the risk of orphaning.

Therefore, understanding the epidemiology of fertility desires of people living with HIV helps clinicians aware of their clients demand ahead of time and gets prepared to plan and implement the preventive modalities for both horizontal and vertical HIV transmission. This is to mean that letting the couples know the potential health risks to themselves and their babies help them make an informed decision, which is also the recommendation of other authors [15,44]. When the fertility desire comes into practice, safer pregnancies practice such as making low preconception viral load using ART and instituting the principles of prevention of mother-to-child transmission of HIV (PMTCT) during pregnancy are proven to be the best protective methods of HIV vertical transmission [44].

These meta-analyses are not without limitations. Firstly, the duration of ART was not taken into consideration. It is known that as the duration of therapy increases, the general well-being of ART patients improves [10,38] and as a result may have more fertility desire, which should be an area of investigation. The reduction in vertical HIV transmission due to ART and safe delivery is also likely to increase the desire for having a biological child. The finding of about 2-fold and 3-fold increment in fertility desires in Brazil [28] and Canada [12], respectively, is another evidence to speculate the probable increment in fertility desire in the years to come.

Secondly, it is difficult to make conclusions on fertility desires taking only four variables (ART, age, sex, education and number of children) as determinants. There are other variables like culture, religion, marriage, income, partner influence, and like, which we were not able to make analyses because of either lack of or unfitness of data for meta-analyses. A systematic review by Nattabi et al. in 2009 also concluded that fertility desires are influenced by a myriad of factors (demographic, health-related, stigma associated, psychosocial and cultural) [18]. Thirdly, because of the sensitiveness of the fertility issue for some society, the respondents may not express their genuine desire. It was reported that most HIV positive women had not discussed their fertility desires with health care providers due to fear of anticipated negative reactions and few attempted found that the environment was unsupportive for open discussion [15,20,22,28]. Fourthly, these meta-analyses included studies mainly from Sub-Saharan Africa and few from developed countries. Thus, the findings of this analysis are unlikely to be representative of the countries where the included studies done.

## Conclusions

Although the fertility desire among childless and the young group was very strong, in general, we realized that quite a significant segment of HIV-infected people has desires for fertility. The reviewed literature also identified a gap in addressing fertility issue in the routine care for HIV positive people. Therefore, including fertility issue as integral part of HIV patient care and counseling can help several of them in their reproductive decision making. Such counseling needs to focus on letting HIV positive people know the risks and methods of prevention while anticipating a pregnancy.

## Competing interests

The authors declare that there is no competing interest. For this analysis, we have not got any financial or technical support.

## Authors’ contributions

YB wrote the whole manuscript and assisted in study selection and data analysis. AB mainly worked on the data analysis. Both authors read and approved the final manuscript.

## Pre-publication history

The pre-publication history for this paper can be accessed here:

http://www.biomedcentral.com/1471-2458/13/409/prepub
